# Remarkable Ionic Conductivity in a LZO-SDC Composite for Low-Temperature Solid Oxide Fuel Cells

**DOI:** 10.3390/nano11092277

**Published:** 2021-09-01

**Authors:** Zhengwen Tu, Yuanyuan Tian, Mingyang Liu, Bin Jin, Muhammad Akbar, Naveed Mushtaq, Xunying Wang, Wenjing Dong, Baoyuan Wang, Chen Xia

**Affiliations:** 1 Key Laboratory of Ferro and Piezoelectric Materials and Devices of Hubei Province, Faculty of Physics and Electronic Science, Hubei University, Wuhan 430062, China; 201911110510866@stu.hubu.edu.cn (Z.T.); 201722110520069@stu.hubu.edu.cn (Y.T.); 201722110520089@stu.hubu.edu.cn (M.L.); 201911110510874@stu.hubu.edu.cn (B.J.); 201817110500024@stu.hubu.edu.cn (M.A.); wangxunying@hubu.edu.cn (X.W.); wenjingd@hubu.edu.cn (W.D.); 2 School of Energy and Environment, Southeast University, No.2 Si Pai Lou, Nanjing 210096, China; 101300031@seu.edu.cn

**Keywords:** SOFCs, composite electrolyte, Li-doped ZnO, high ionic conductivity, interfacial conduction

## Abstract

Recently, appreciable ionic conduction has been frequently observed in multifunctional semiconductors, pointing out an unconventional way to develop electrolytes for solid oxide fuel cells (SOFCs). Among them, ZnO and Li-doped ZnO (LZO) have shown great potential. In this study, to further improve the electrolyte capability of LZO, a typical ionic conductor Sm_0.2_Ce_0.8_O_1.9_ (SDC) is introduced to form semiconductor-ionic composites with LZO. The designed LZO-SDC composites with various mass ratios are successfully demonstrated in SOFCs at low operating temperatures, exhibiting a peak power density of 713 mW cm^−2^ and high open circuit voltages (OCVs) of 1.04 V at 550 °C by the best-performing sample 5LZO-5SDC, which is superior to that of simplex LZO electrolyte SOFC. Our electrochemical and electrical analysis reveals that the composite samples have attained enhanced ionic conduction as compared to pure LZO and SDC, reaching a remarkable ionic conductivity of 0.16 S cm^−1^ at 550 °C, and shows hybrid H^+^/O^2^^−^ conducting capability with predominant H^+^ conduction. Further investigation in terms of interface inspection manifests that oxygen vacancies are enriched at the hetero-interface between LZO and SDC, which gives rise to the high ionic conductivity of 5LZO-5SDC. Our study thus suggests the tremendous potentials of semiconductor ionic materials and indicates an effective way to develop fast ionic transport in electrolytes for low-temperature SOFCs.

## 1. Introduction

As a quintessential high-efficient and low-emission energy conversion technology, solid oxide fuel cells (SOFCs) have the feature of converting the chemical energy of fuels directly into electricity using a flexible selection of fuels [1]. However, the current commercialization process of SOFC is still restricted by its excessively high operating temperature, as the ionic transport in electrolytes and the catalytic activity of cathode that are linked to the power output and lifetime of fuel cells are thermally driven processes. For instance, the most frequently used electrolyte by far, Y_2_O_3_-stabilized ZrO_2_ (YSZ) strictly requires high temperature over 700 °C to reach a sufficient ionic conductivity to run the fuel cell [2,3,4]. For addressing this challenge, extensive efforts have been made to reduce the operating temperature of SOFCs to a lower range of 400–600 °C [5]. Unfortunately, along with the decrease of temperature, the ohmic polarization of cell invariably presents exponential growth, while the catalytic activity of cathode turns sluggish, thus resulting in severe power losses in the SOFCs. Therefore, developing low-temperature (LT) electrolytes and cathodes that can capably work at 400–600 °C is highly desired for SOFCs.

With respect to the research and development of desirable electrolytes, recent studies have revealed that the use of BaCeO_3_ and BaZrO_3_-based proton conductors, oxygen-deficient semiconductors, composite materials, and the reduction of YSZ thickness by thin film techniques are feasible approaches, which result in modest decreases in the operating temperature of SOFCs down to 500–700 °C [6,7,8,9,10,11]. Particularly, semiconductor ionic materials and composite have been used to develop electrolytes to enable high ionic conductivity at low temperatures of 300–500 °C. These semiconductor electrolytes realize their electrolyte functionality via various semiconductor properties and conduction mechanisms, which are different from that of traditional electrolytes such as YSZ and Sm_0.2_Ce_0.8_O_1.9_ (SDC) that primarily rely on cation substitution to create oxygen vacancies. For example, a layer-structured semiconductor Li_x_Al_0.5_Co_0.5_O_2_ with interlamination diffusion of protons was applied in SOFC as the electrolyte layer, with good ionic conduction of 0.1 S cm^−1^ at 500 °C [9]; a semiconductor electrolyte SmNiO_3_ with high initial ionic and electronic conductivity was demonstrated in SOFC via a filling-controlled Mott transition effect, achieving peak power output of 225 mW cm^−2^ and sufficient open-circuit voltage (OCV) at 500 °C [10]. Inspired by these studies, a sequence of multifunctional semiconductors were then taken into consideration for electrolyte use by virtue of their surface oxygen defect diffusion and energy band alignment effect, such as TiO_2_, α-Fe_2_O_2-δ_, and SrTiO_3_ [11,12,13]. Our recent findings showed that nano-sized ZnO has a hybrid proton and oxygen ion conductivity of 0.09 S cm^−1^ at 550 °C with low activation of 0.7 eV, when applied as the electrolyte in an SOFC with Ni_0.8_Co_0.15_Al_0.05_LiO_2-δ_ (NCAL) symmetrical electrodes, the ZnO can achieve promising fuel cell power outputs of 158–482 mW cm^−2^ at 450–550 °C [14]. Furthermore, its derivative Li-doped ZnO (LZO) was developed using a doping method, which revealed distinct promotion on the electrode reaction activities of fuel cells by right of its electronic conduction and Li+ diffusion, leading to diminished polarization resistances, despite the fact that the ionic conduction was not improved [15]. These studies indicate the promising ionic and catalytic properties of ZnO-based materials for electrochemical uses in addition to their well-known optoelectronic properties and thermal stability [16,17,18,19].

In order to further improve the electrolyte capability of LZO, the aforementioned composite strategy which has been frequently used in developing fast ionic conduction is considered herein. With respect to the composite electrolytes, typical representatives include the ceria-salt system which is commonly made of doped ceria and alkaline carbonate, and the semiconductor-ionic system consisting of semiconductor and ionic conductors [20,21,22,23,24]. As reported, by incorporating carbonate (25 wt% Na_2_CO_3_) into SDC to form a core-shell structure, the ionic conductivity of SDC can be dramatically improved due to interfacial dual ionic conduction [21]. This ionic enhancing behavior has also been discovered in semiconductor-ionic composites, for instance, an extraordinarily high ionic conductivity of 0.188 S cm^−1^ was detected in a composite of La_0.6_Sr_0.4_Co_0.2_Fe_0.8_O_3-δ_ (LSCF) and Ca_0.04_Ce_0.80_Sm_0.16_O_2-δ_ (SCDC) at 600 °C, while a similar high ionic conductivity was also observed in a composite based on NCAL and Ce_0.8_Sm_0.2_O_2-δ_-Na_2_CO_3_ (NSDC) [22,23]. These high ionic conduction examples are widely regarded as a result of hetero-interface superionic transport, since highly disordered oxygen plane can be created at semiconductor/ionic conductor interface as reported by previous study when Garcia-Barriocanal et al. detected a colossal ionic conductivity of ~0.1 S cm^−1^ at 200 °C at the YSZ/SrTiO_3_ interface [25]. These semiconductor-ionic composites cast a new light on SOFC electrolytes and provide a underlying approach for boosting the ionic conduction of the studied LZO.

Therefore, in the present study, we introduce various ratios of SDC into LZO to develop semiconductor-ionic composites for high-performance SOFC electrolyte applications. The LZO-SDC composites are studied in terms of crystalline-structural, morphological, electrical, and electrochemical characterizations. The best-performing sample demonstrates a favorable electrolyte function with improved ionic conduction and fuel cell performance as compared to single LZO. Further investigation gains insight into the interface of LZO-SDC to study its conducting behavior. Our approach thus provides a feasible way to develop LT electrolyte for SOFCs.

## 2. Experimental Section

The LZO used in this study was synthesized by a co-precipitation method. The precursors were analytical grade zinc nitrate hexahydrate (Zn(NO_3_)_2_·6H_2_O, Sigma Aldrich, Shanghai, China, 99.5%), lithium nitrate (LiNO_3_, Sigma Aldrich, Shanghai, China 99%) and sodium carbonate (Na_2_CO_3_, Sigma Aldrich, Shanghai, China 99%) all used without further purification. Zn(NO_3_)_2_·6H_2_O and LiNO_3_ were dissolved in deionized water to form a 1 M solution with a molar ratio of metal ions of Li:Zn = 2:8. The resulting solution was vigorously stirred at 600 rpm and heated at 60 °C for 0.5 h. Subsequently, under continuous magnetic stirring for 1 h, a 1 M Na_2_CO_3_ solution was added as a precipitation agent to the above nitrite solution to form a white precipitate. The resulting precipitate was then filtered and washed with deionized water for three times to remove some surface impurities, followed by drying in air overnight at 120 °C. Finally, the dried precursor was sintered in air at 600 °C for 2 h to obtain a white powder, which was then collected and ground in an agate mortar to form the final product of Li_0.2_Zn_0.8_O.

The SDC is also synthesized by the co-precipitation method. Under continuous stirring, the stoichiometric precursors Ce(NO_3_)_3_·6H_2_O (Sigma-Aldrich, Shanghai, China 99.5%) and Sm(NO_3_)_3_·6H_2_O (Sigma-Aldrich, Shanghai, China 99.5%) were dissolved in deionized water to form a 1 M solution. Then, according to the molar ratio of metal ion:carbonate ion = 1:1.5, 1 M of Na_2_CO_3_ solution as a precipitant was added dropwise to the above nitrate solution to indice precipitation. Subsequently, the precipitate was repeatedly filtered and washed to remove some surface impurities, and then dried at 120 °C in air for 24 h. Finally, the material was sintered in air at 800 °C for 4 h before being sufficiently ground to obtain SDC powder.

The LZO-SDC composite samples were prepared using a solid-state mixing procedure, by blending the resultant LZO and SDC powders in various mass ratios (7:3, 5:5, 3:7) in a ball mill for 8 h under a rotation speed of 250 r min^−1^ using 10 mm-sized agate balls. Subsequently, the mixtures were sintered in air at 650 °C for 2 h before complete grinding to obtain the powder samples of 7LZO-3SDC, 5LZO-5SDC, and 3LZO-7SDC.

A commercial Ni_0.8_Co_0.15_Al_0.05_LiO_2-δ_ (NCAL) was used as symmetrical electrode in our SOFC devices in a form of NCAL-pasted Ni-foam (NCAL-Ni). The Ni-foam with porosity of 50–90 % and pore size of ~0.1 mm is purchased from Huirui Wire-mesh Company (Shengzhen, China). The NCAL-Ni were fabricated by mixing NCAL powders with terpineol solvent to form a slurry, which was then pasted on Ni foam and desiccated at 120 °C for 0.5 h to form NCAL-Ni electrode pieces. To assemble LZO-SDC SOFCs, the prepared LZO-SDC sample was sandwiched between two NCAL-Ni electrodes and pressed under a uniaxial load of 200 MPa into a pellet, followed by brushing the two electrode surfaces with silver paste (Kunshan Yosoar New Materials Co., Ltd., Kunshan, China) as current collector. The obtained cell pellet is in a configuration of NCAL-Ni/LZO-SDC/NCAL-Ni with thickness of ~0.6 mm and active area of 0.64 cm^2^. Besides, for our comparative study, a simplex LZO electrolyte powder was also ball milled and sintered in the same ways to fabricate a NCAL-Ni/LZO/NCAL-Ni SOFC by the same assembly procedure. All these fuel cells were mounted into testing fixture and experienced an online sintering in air at 600 °C for 0.5 h prior to operation and testing.

The crystal structures of the prepared materials were examined by a D8 Advanced X-ray diffractometer (XRD, Bruker, Ettlingen, Germany) with Cu Kα (λ = 1.54060 Å) source (tube voltage of 45 kV, working current of 40 mA). The morphology of the materials along with the elemental composition were analyzed via a field emission scanning electron microscope (FE-SEM, JEOL JSM7100F, Tokyo, Japan) with energy dispersive X-ray spectrometer (EDS). A transmission electron microscope (TEM, JEOL JEM-2100F) operating under accelerating voltage of 200 kV was employed to capture the micro-structure of the best-performing sample. Furthermore, X-ray photoelectron spectroscopy (Escalab 250 Xi, Thermo Fisher Scientific, Gloucester, UK) was used to measure X-ray photoelectron spectroscopy (XPS) for checking the surface properties of the LZO-SDC powders. The base pressure was about 5 × 10^−^^10^ mbar and the pass energy is 100 eV with binding energy step size of 0.5 eV. Raman spectra were carried out on an NT-MDT Raman spectrometer (Moscow, Russia) equipped with an LCM-S-111 laser at room temperature. The excitation source is a 532 nm solid-state laser, and the laser power on the sample is about 20 mW.

The materials were fabricated into disc pellets with an active area of 0.64 cm^2^. The fuel cells were operated in a temperature range of 500–550 °C with dry pure hydrogen (Wisco Oxygen Co., Ltd., Wuhan, China) and ambient air as fuel and oxidant (120–140 mL min^−1^), respectively. The current-voltage (I-V) and current-power (I-P) characteristics of fuel cells were measured by a IT8511 electronic load (ITECH Electrical Co, Ltd, Nanjing, China) with a IT7000 software (version 1.0; ITECH Electrical Co, Ltd.; Nanjing, China, 2015) to record the data and adjust the scan speed. The electrochemical impedance spectra (EIS) of the fuel cells were measured by using a Gamry Reference 3000 electrochemical workstation (Gamry Instruments, Warminster, PA, USA), which were carried out under open circuit voltage (OCV) mode with applied frequency of 0.1–10^5^ Hz and an alternating current (AC) voltage of 10 mV in amplitude. The measured results were fitted by the ZSimWin software (version 2.0; PerkinElmer Instruments; Ann Arbor, MI, USA, 2001). The DC conductivity measurements of the samples were carried out using a digital sourcemeter (Keithley 2400, Cleveland, OH, USA) with two electrodes in various atmospheres of ambient air, pure nitrogen, and hydrogen. The electrical conductivities of LZO, 3LZO-7SDC, 5LZO-5SDC and 7LZO-3SDC were measured based on the disc pellets of the four samples, using a Gamry Reference 3000 electrochemical workstation under OCV mode with applied frequency of 0.1–10^5^ Hz with an AC voltage of 10 mV in amplitude in fuel cell operating atmosphere. Four disc pellets with active area of 0.64 cm^2^ for the samples were prepared by pressing the sample powders under a uniaxial load of 200 MPa, followed by brushing platinum paste on the both surfaces for EIS test.

## 3. Results and Discussion

### 3.1. Crystalline Structure and Morphology

**Figure 1:**
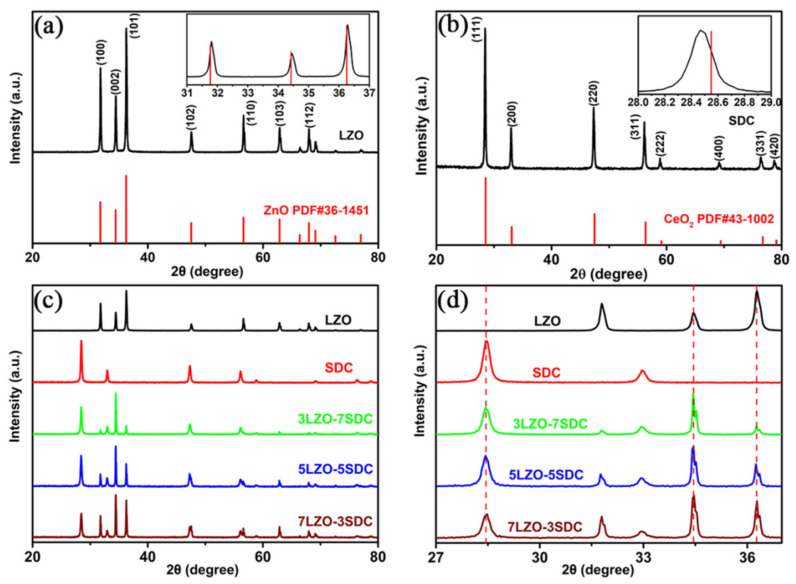
XRD patterns of the prepared (**a**) LZO, (**b**) SDC, and (**c**) LZO-SDC composites; (**d**) Enlarged XRD patterns of the LZO-SDC composites.

Figure 1a shows the XRD patterns of the prepared LZO. Typical diffraction peaks of LZO can be indexed to the (100), (002), (101), (102), (110), (103) and (112) planes, matching well to those of the standard ZnO with hexagonal wurtzite structure (PDF No. 36-1451). Besides, it is evident that no peaks of the metal nitrates can be observed in the pattern of LZO, and there are no extra signal corresponding with the Zn, Li, or Li_2_O related secondary phases, reflecting that Li is completely incorporated into the crystal lattice of ZnO through occupying an interstitial site or a substitution site of Zn. The Li-doping cause a very slight peak shift of Li-doped ZnO to higher 2θ angle as compared with the standard diffraction peaks of ZnO in Figure 1a. This should be attributed to the approximately same ionic radii of Li^+^ (0.73 Å) and Zn^2+^ (0.74 Å) [26,27].

Figure 1b shows the XRD patterns of the prepared SDC. The diffraction peaks corresponding to (111), (200), (220), (311), (222), (400), (331) and (420) planes match well to the XRD of cubic fluorite CeO_2_ (PDF No. 43–1002) without any secondary phase peaks, while revealing a slight shift to smaller 2θ angle compared to that of standard CeO_2_. This suggests that the obtained SDC is a Sm-doped CeO_2_, since the substitution of Ce^4+^ (ionic radius 0.97 Å) by Sm^3+^ (ionic radius 1.079 Å) would enlarge the crystal lattice and result in a left shift according to the Bragg’s law [28,29]. According to the XRD results, the d spacings of (002) plane for LZO and (111) plane for SDC is calculated as 0.2602 and 0.3133 nm, respectively. On basis of which, the lattice parameters can be also calculated as *a* = *b* = 0.3241 nm, *c* = 0.5204 nm for LZO, smaller than that of ZnO (PDF No. 36-1451), and as *a* = *b* = *c* = 0.5430 nm for SDC, which is much bigger than that of CeO_2_ (PDF No. 43-1002). This confirms the successful doping of Li and Sm in LZO and SDC samples, respectively. According to Vegard‘s law, the stoichiometry of the prepared LZO be also estimated to obtain the chemical formula of LZO, which is Li_0.16_Zn_0.84_O. This means there should be slight Li evaporation during sintering process of LZO. Moreover, Figure 1c illustrates the XRD patterns of the three LZO-SDC composite samples in comparison with pure LZO and SDC. All diffraction peaks of the three sample can be assigned to either LZO or SDC, evidencing that the composite has a heterostructure feature with combinative phase structures of LZO and SDC. No obvious peak shift or appearance of new phases can be observed, as verified by the enlarged XRD pattern in Figure 1d. According to the (101) and (002) planes of LZO, and (111) plane of SDC in Figure 1d, the cell parameters of the LZO and SDC in 3LZO-7SDC, 5LZO-5SDC, and 7LZO-3SDC were also calculated. It is found the lattice parameters of LZO in the three samples are almost the same as *a* = *b* = 0.3240 nm, *c* = 0.5204 nm, while that for SDC in the three samples are all around *a* = *b* = *c* = 0.5433 nm. These results are extremely close to the values of LZO and SDC before sintering process, which excludes the possibility of unit cell volume variation of LZO and SDC in the three samples before and after sintering, indicating no chemical interaction between the two phases. Particularly, it suggests that there was no distinct evaporation of Li during the sintering process. Besides, several shoulder peaks appear in the XRD of three LZO-SDC composites, which is probably due to the presence of two radiation (Cu Kα1, Kα2) during tests with different characteristic energies of X-ray photons. The XRD results manifest that LZO and SDC co-exist in the composite samples without any chemical interaction between the two phases during material synthesis procedure [30,31].

**Figure 2:**
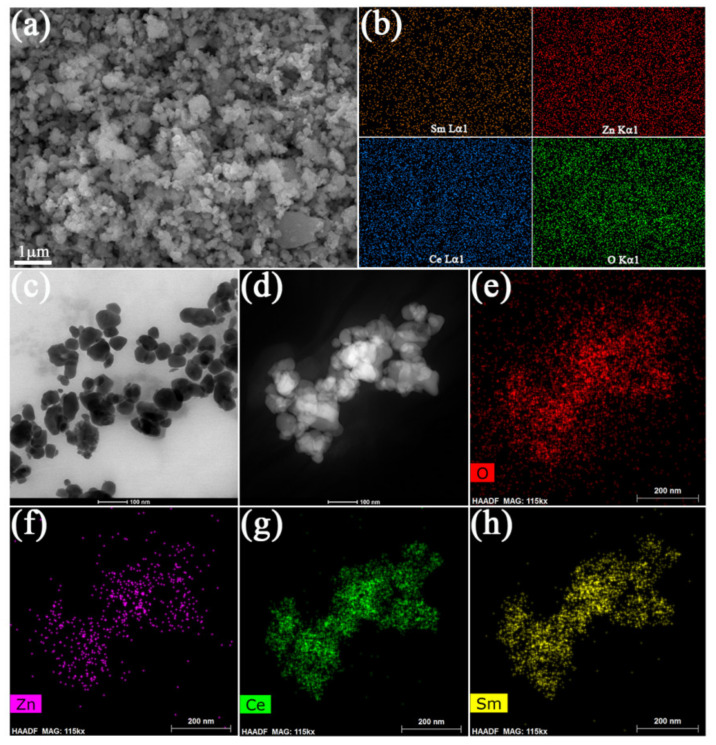
(**a**) SEM image and (**b**) EDS mappings of the 5LZO-5SDC powder; (**c**) bright-field and (**d**) dark-field TEM images of 5LZO-5SDC, and the elemental mappings of (**e**) O, (**f**) Zn, (**g**) Ce, (**h**) Sm based on the dark-field TEM image.

Figure 2a,b show the SEM image and EDS mappings of the 5LZO-5SDC sample. As can be seen, the 5LZO-5SDC powder is made of nanoparticles with a size of ~100 nm. The elemental distributions of Sm, Zn, Ce, and O are quite homogeneous, suggesting uniform dispersion of LZO and SDC in the composite. Figure 2c provides a bright-field TEM image to show the grain/particle shape and size. Grains and agglomerated particles with irregular shapes are clearly observed, with size of 50–100 nm. Figure 2d–f further show dark-field TEM images to depict the particle contacts and the corresponding element mappings. In Figure 2d, the nano-sized particles are closely contacted. From the mapping results, it is known that the elements from LZO and SDC are distributed evenly in the TEM image. This manifests that the semiconductor and ionic phases have formed massive hetero-interfaces in the composite sample, which are potential to create highly disordered oxygen plane or space charge regiond at the interfaces to enable fast oxygen vacancy diffusion of ions [25,32,33]. This conjecture is further verified in the following section by electrochemical and electrical analyses.

### 3.2. Electrochemical Performance

The developed LZO and LZO-SDC composites were applied in SOFCs for comparison to assess the feasibility of our approach. Fuel cell electrochemical performances were measured in the low temperature range of 500–550 °C after stabilizing the OCVs of the fuel cells.

**Figure 3:**
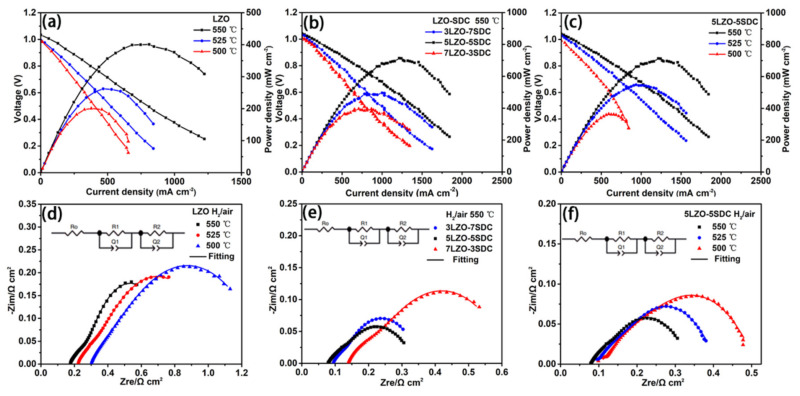
Electrochemical performance of (**a**) LZO SOFC at 500–550 °C, (**b**) LZO-SDC SOFCs with various compositions at 550 °C, and (**c**) 5LZO-5SDC SOFC at 500–550 °C; the corresponding EIS for (**d**) LZO SOFC, (**e**) LZO-SDC SOFCs with various compositions, and (**f**) 5LZO-5SDC SOFC measured in H_2_/air.

Figure 3a presents the I-V and I-P characteristics for the LZO SOFC. As the temperature increases from 500 °C to 550 °C, the power density rises from 203 mW cm^−2^ to 406 mW cm^−2^. In Figure 3b, the performances of fuel cells with different composite ratios of LZO-SDC electrolytes acquired at 550 °C are presented, and an obvious dependence of power output on the LZO/SDC ratio can be observed. Among the three electrolytes, the 5LZO-5SDC represents the highest fuel cell power density of 713 mW cm^−2^ at 550 °C, while the power density of 3LZO-7SDC and 7LZO-3SDC are 496 mW cm^−2^ and 396 mW cm^−2^, respectively. The performance of 7LZO-3SDC cell is comparable with that of LZO cell. In Figure 3c, the performance of the 5LZO-5SDC fuel cell at various temperatures is further shown. In our previous study, the SDC electrolyte-based SOFC with the same NCAL-Ni electrodes exhibited a peak power density of 389 mW cm^−2^ at 550 °C [34]. Compared with simplex LZO and SDC fuel cells, the power density of 5LZO-5SDC fuel cell is remarkably improved, while the fuel cells based on other two composites reveal slightly progressive or comparable power outputs. Besides, it is also worth noting that the best-performance fuel cell demonstrates a promising power density of 375 mW cm^−2^ at 500 °C that is close to the power density of the LZO cell at 550 °C, which means the composite approach lowered the operating temperature of LZO fuel cell without any distinct performance sacrifice.

In addition to the appreciable power densities, high OCVs were also achieved by the fuel cells. For a conventional SOFC, semiconductor LZO is not suitable to be used as the electrolyte layer, since the electrons produced at the anode could transfer to the semiconductor electrolyte to induce short-circuiting issues [35,36], and the used SDC is commonly reduced in a H_2_ rich atmosphere, resulting in unfavorable deterioration of OCV. However, these concerns have been eliminated in our case as proved by the high OCVs, which are close to or even higher than 1 V as shown in Figure 3. This can be attributed to a Schottky junction effect based on metal-semiconductor contacts between Ni/Co (reduced NCAL) and LZO-SDC, which has been reported before with a function of blocking electron passage from anode to semiconductor electrolyte by the Schottky barrier when operating the fuel cells [14,33,34].

Furthermore, in order to study the electrochemical characteristics of the assembled fuel cells, EIS measurements were performed under H_2_/air conditions. The measured Nyquist curves at different temperatures are shown in Figure 3d–f. After using ZSimDemo to fit the experimental data, the simulated EIS curves are plotted together with the experimental results, as can be seen in Figure 3d–f. The corresponding equivalent circuit model *R*_o_(*R*_1_*Q*_1_)(*R*_2_*Q*_2_) for fitting is also presented as insets in these figures, where *R*_o_ is regarded as the ohmic resistance of cell including both ionic and electronic resistance contribution, and the *R_1_* and R_2_ always denote charge transfer and mass transfer processes of the electrode activity. The *R_o_* can be determined by the intercept of the real axis at high frequencies. The sum of *R_1_* and *R_2_* is defined as the electrode polarization resistance (*R_p_*), which is related to the basic electrode reaction [37,38]. The corresponding characteristic capacitance (*C_i_*) for each process can be calculated by equation (1), where Q is a constant phase element (CPE), which represents a non-ideal capacitor, *R_i_* (*i* = 1, 2) is the resistance, and n represents the similarity between CPE and an ideal capacitor; when *n* = 1, CPE can be considered as an ideal capacitor [39,40]. Under normal circumstances, *n* is less than 1. Each arc (*R_i_Q_i_*) (*i* = 2,3) should be attributed to the corresponding process according to the value of its characteristic capacitance *C_i_*:

(1) $$C_{i}=\frac{{\left(R_{i}Q_{i}\right)}^{1/n}}{R_{i}}$$

According to the EIS in Figure 3d,f, it is found the 5LZO-5SDC cell has almost half the *R_o_* and *R_p_* as compared to the LZO cell. This means the composite sample contributes not only improved ionic conductivity, but also facilitated electrode activity to the cell, which should be a result of high ionic conduction of 5LZO-5SDC to enable fast oxygen ion migration to the triple phase boundary, resulting in rapid oxygen oxidation reaction. In Figure 3e, the EIS of the three LZO-SDC SOFCs tested at 550 °C are plotted for comparison. The corresponding simulated parameters are listed in Table 1. It can be found the *R_o_* of 5LZO-5SDC cell is smaller than other two cells, as an indication of higher ionic conductivity the 5LZO-5SDC electrolyte. In light of the values of C_i_, the *R_1_* and *R_2_* can be explicitly assigned to charge transfer and mass transfer resistance. Thus the *R_p_* of the three cells can be obtained as 0.2479, 0.2561, and 0.4808 Ω cm^2^, respectively. Due to the lower ohmic and polarization resistence, the 5LZO-5SDC cell showed higher performance. In addition, the resistance parameters (*R_o_* and *R_p_*) of 5LZO-5SDC cell acquired from EIS were further compared to the previously reported SOFCs based on LZO, SDC, SrTiO_3_-SDC, LSCF-SCDC, NCAL-NSDC electrolytes using the same NCAL-Ni electrodes [14,22,23,34]. As summarized in Table 2, the 5LZO-5SDC cell reveals lower *R_o_* and *R_p_* than those of other five cells, implying higher ionic conductivity and higher electrode reaction activity of the 5LZO-5SDC cell. These comparison results reflect the ionic conduction of simplex electrolyte can improved by forming a semiconductor-ionic composite, and LZO seems to be a more proper partner for SDC to form the composite electrolyte.

The above performance and EIS results verify that the 5LZO-5SDC has superior power output and electrolyte capability than simplex LZO and other composites, and this is most probably due to the enhanced ion conductivity of LZO-SDC through interface conduction. Hence, the conductivity and interface property of the best-performing sample are further investigated in following two sections.

### 3.3. Electrical Conductivity

**Figure 4:**
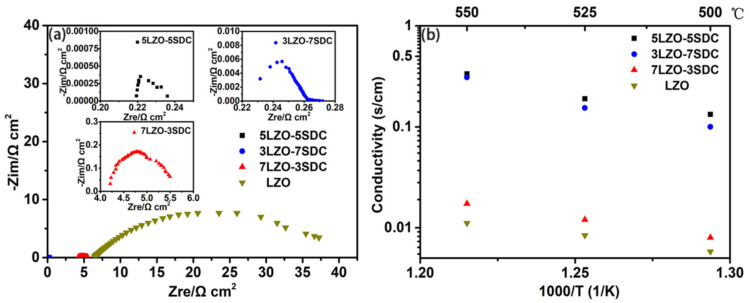
Typical impedance spectra of (**a**) LZO, 7LZO-3SDC, 5LZO-5SDC and 3LZO-7SDC pellets measured at 550 °C in H_2_/air, and (**b**) the electrical conductivity of LZO, 5LZO-5SDC, 7LZO-3SDC, and 3LZO-7SDC attained from EIS results.

Figure 4a provides the EIS curves of LZO, 7LZO-3SDC, 5LZO-5SDC and 3LZO-7SDC pellets tested at 500–550 °C in H_2_/air. It can be found the introduction of SDC into LZO significantly reduce the ohmic resistance of EIS. On basis of the EIS results, the electrical conductivities of LZO and the three LZO-SDC composites can be obtained, as shown in Figure 4b. The conductivity of the best-performing sample 5LZO-5SDC reaches a value of 0.335 S cm^−1^ at 550 °C, which is slightly higher than that of 3LZO-7SDC (0.31 S cm^−1^). Both electrical conductivities of the two samples are one order of magnitude higher than that of 7LZO-3SDC and LZO. Even though this conductivity order is accordant with the order of fuel cell performance, we still think the values for 5LZO-5SDC and 3LZO-7SDC are excessively high as compared to LZO and 7LZO-3SDC. One possible reason leading to this result can be that high electronic conduction was induced into the composites because of partial reduction of SDC in H_2_ when the mass ratio of SDC is more than 30 wt%. Unlike conventional electrolytes, the 5LZO-5SDC composite may involves considerable electron conduction. This makes it imprecise to use only EIS technique for ionic conductivity assessment, as electrons would partially affect the ohmic resistance and grain-boundary resistance of EIS. Therefore, in the current work, another method which has been recently proposed to evaluate the conduction of semiconductor electrolytes is used to study the ionic conductivity of LZO-SDC composites [41,42]. In this method, the linear central region of a polarization curve reflecting ohmic polarization loss (Δ*V_ohm_*) of the tested cell, which consists of contributions from electrolyte and electrode components. Considering that the NCAL electrode material has a high triple charge (H^+^/O^2−^/e^−^) conductivity of around 10 S cm^−1^ at 550 °C [43,44], the electronic resistance of electrodes should be negligible in contrast to the ionic resistance of LZO and LZO-SDC electrolytes. In this regard, the Δ*V_ohm_* obtained from polarization curve can be almost all attributed to the ionic resistance of electrolyte. Thus, the linear central region of a polarization curve was used to obtain the area specific resistance of electrolyte (*R_ASR_*) by *R_ASR_ =* Δ*V_ohm_/*Δ*I_ohm_* in terms of Δ*V_ohm_* and the corresponding current drop (Δ*I_ohm_*) [45]. In this way, the ionic conductivity (*σ_i_*) of LZO and the LZO-SDC composites electrolytes can be calculated according to:

(2) $$\sigma_{i}=\frac{L}{R_{ASR}\times S}=\frac{\Delta I_{ohm}\times L}{\Delta V_{ohm}\times S}$$

**Figure 5:**
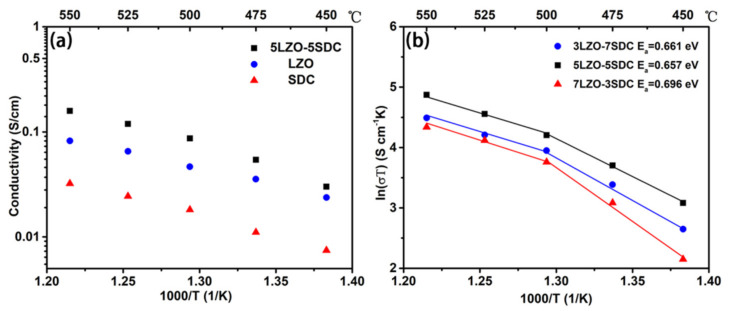
(**a**) Ionic conductivity plot of LZO and 5LZO-5SDC composites as a function of 1000/T obtained from I–V polarization curves at 450–550 °C for comparison with reported SDC; (**b**) activation energy (*E_a_*) plots of LZO-SDC composites as a function of 1000/T with *E_a_* values in the range of 500–550 °C.

The obtained values of σ_i_ as a function of temperatures are summarized in Figure 5a. As can be seen, the individual LZO exhibits an ionic conductivity of 0.08 S cm^−1^ at 550 °C, while the 5LZO-5SDC reveal a higher ionic conductivity 0.16 S cm^−1^. The ionic conductivity of SDC reported in our previous study was also plotted in Figure 5a for comparison [34]. A significant enhancement of ionic conductivity is observed in the composite electrolyte after SDC is introduced into LZO, substantiating that the used composite approach is feasible to enable remarkable ionic conductivity in LZO-SDC system. Besides, the ionic conduction of the three composite samples were also calculated and presented in terms of Arrhenius curve, as shown in Figure 5b. The activation energy of ionic conduction can be calculated according to the Arrhenius Formula (3), where T is the absolute temperature, A is the pre-exponential factor, is the activation energy, and k represents the Boltzmann constant [46]. It is found the three samples have almost same activation energy values of the ionic conduction in fuel cell operating temperature range of 500–550 °C, whereas the 5LZO-5SDC shows the highest ionic conductivity. The activation energies are in the range of 0.657–0.696 eV, which is close to the normal activation energy of proton diffusion. This means the composites are very likely hybrid oxygen ion and proton conductors:

(3) $$\sigma T=Aexp\left[-E_{a}/\left(kT\right)\right]$$

**Figure 6:**
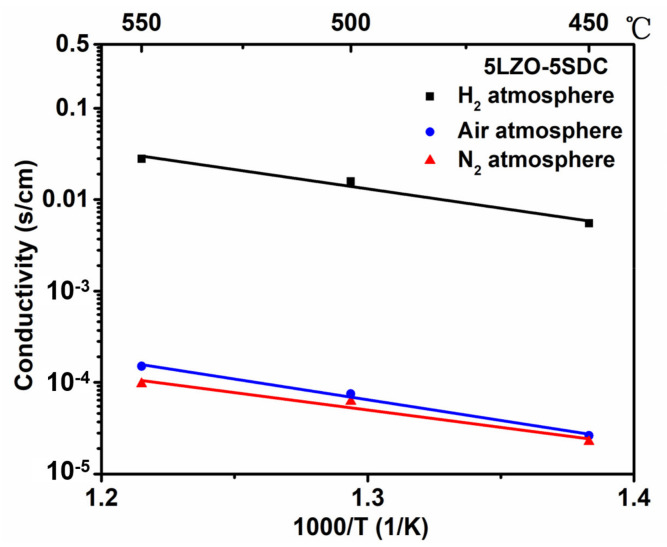
The electrical conductivity of 5LZO-5SDC at 450–550 °C acquired in air, nitrogen, and hydrogen atmospheres.

To further study the conducting behavior of the composite, the DC electrical conductivity of the best-performing sample, 5LZO-5SDC was measured in ambient air, pure nitrogen, and pure hydrogen atmospheres, respectively. A stationary voltage of 0.5 V was applied to the pellet, and the current-time curve was recorded until the fuel cell is stabilized. It is observed the current is extremely low regardless of measuring in an air atmosphere or a nitrogen atmosphere. Figure 6 shows the electrical conductivity results of 5LZO-5SDC tested in different atmosphere at 450–550 °C, which are also summarized in Table 3. The corresponding activation energies were calculated and shown in Table 3, which suggest that the electrical conductivities measured in N_2_, air, and H_2_ are electronic conductivity, mixed oxygen-ion and electronic conductivity, and proton conductivity, respectively. In N_2_, the conductivity that only contains electronic contribution shows a negligible value in the 10^−5^ S cm^−1^ level, which is favourable for the composite sample to be used as an electrolyte. In air, the conductivity, which includes the major contribution of oxygen ions and minor contribution of electrons, reaches a value of 2.6 × 10^−5^–1.5 × 10^−4^ S cm^−1^ at 450–550 °C, implying the low oxygen-ion conductivity of 5LZO-5SDC in air. In H_2_, the proton conductivity of 5LZO-5SDC achieve values of 0.0055–0.028 S cm^−1^ at 450–550 °C, showing two order of magnitude enhancement as compared to the result in air. This means the composite tends to be more highly ion-conducting in fuel cell operating atmosphere, as former studied has pointed out that ZnO owns few oxygen vacancies in air, but it can gain significantly enriched surface oxygen vacancies after treated in reducing atmosphere [47].

**Figure 7:**
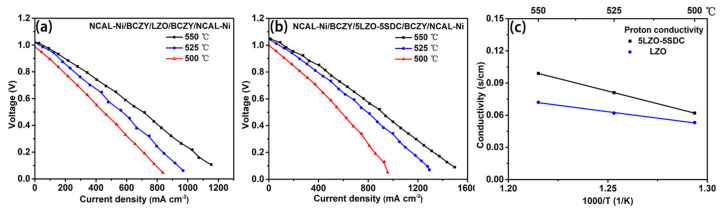
I-V polarization curve of the two O^2−^/e^−^ filtering fuel cells based on (**a**) LZO and (**b**) 5LZO-5SDC, and (**c**) the corresponding proton conduction estimated based on (**a**,**b**).

In view of these conducting behaviors, it is regarded that the LZO and LZO-SDC composites possess hybrid oxygen ion and proton (O^2−^/H^+^) conduction with dominated H^+^ transport. To verify the conjecture, two O^2−^/e^−^ filtering fuel cells in configurations of *NCAL-Ni/BCZY/LZO/BCZY/NCAL-Ni* and *NCAL-Ni/BCZY/5LZO-5SDC/BCZY/NCAL-Ni* were fabricated by using BaCe_0.5_Zr_0.35_Y_0.15_O_3-δ_ (BCZY) as the filtering layer. This filtering approach has been reported in previous studies to measure the specific nature of ionic transport via getting rid of the influence of other carriers [22,42]. Since proton conductor BCZY has high H^+^ but negligible O^2−^/e^−^ conduction, the BCZY/electrolyte/BCZY trilayer would only allow H^+^ to transit to contribute to the fuel cell current output while screening O^2−^. By this means, only proton conduction of the electrolyte contributes to the current density of the fuel cell when operate the two filtering fuel cells, and thus the proton conductivities of LZO and 5LZO-5SDC can be determined from the Ohmic polarization region of I-V curve. As shown in Figure 7a,b, the typical I-V characteristics of the two O^2−^/e^−^ filtering fuel cells were measured at 500–550 °C, demonstrating high current densities. This confirms the appreciable proton conducting capability of LZO and 5LZO-5SDC. According to the polarization curve, the proton conduction of the two electrolytes can be estimated. As shown in Figure 7c, the proton conductivity of LZO is 0.05–0.07 S cm^−1^ at 500–550 °C while that of 5LZO-5SDC reaches 0.06–0.1 S cm^−1^ in the same temperature range. Compared with the results in Figure 5, both proton conductivities of LZO and 5LZO-5SDC fill 60–80% portion of their total ionic conduction, verifying the hybrid H^+^/O^2−^ conduction feature of the two electrolyte materials with predominant H^+^ conduction.

### 3.4. Interface Property and Conduction

**Figure 8:**
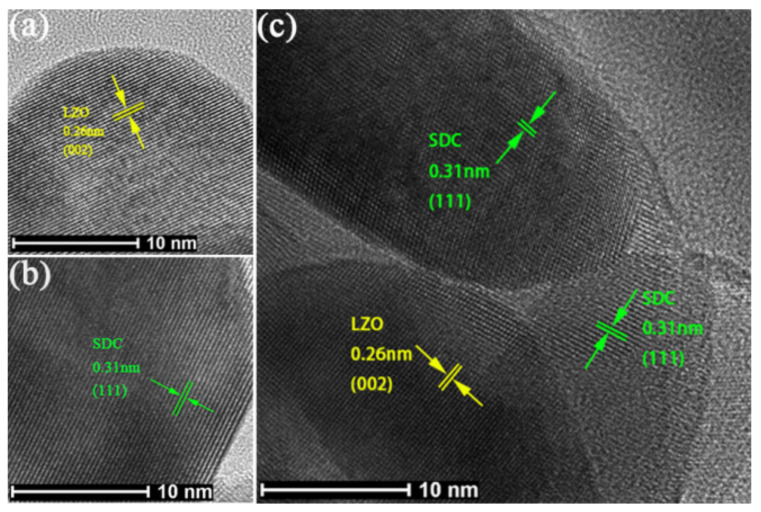
HR-TEM images showing the grains of (**a**) LZO and (**b**) SDC with well-defined crystalline lattice; (**c**) a typical hetero-interface between LZO and SDC grains.

In this section, the interface features of the best-performing sample is investigated. The detailed micro-structure of 5LZO-5SDC was investigated first by high-resolution TEM (HR-TEM). As shown in Figure 8a,b, the grains of LZO and SDC present well-defined crystalline lattice fringes with d-spacings of 0.26 nm and 0.31 nm, respectively. These values are in good agreement with the calculated of d-spacing results (0.2602 nm, 0.3133 nm) from XRD as indicated in Section 3.1, and in line with the reported results in previous literature [48,49]. Furthermore, a typical interface HR-TEM image showing the contacts among three grains is displayed in Figure 8c, in which the clear fringes with lattice spacings of 0.26 and 0.31 nm corresponding to the (002) plane of LZO and the (111) plane of SDC are observed, respectively. This identifies the hetero-interface between the two LZO and SDC phases with different lattice constants, which holds great potential to enable improved ionic conduction of the composite material via interface conduction, as lattice strains and misfit dislocations associated with the generation of oxygen defects are often created at hetero-interface regions [25,34,50].

**Figure 9:**
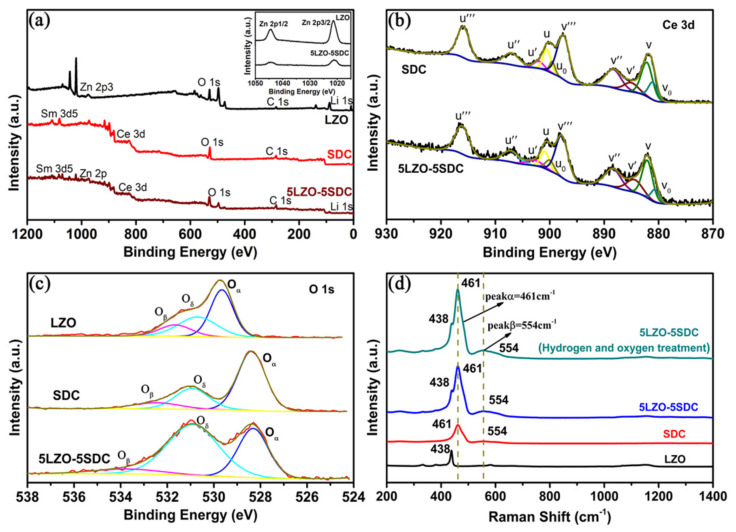
XPS results for the LZO, SDC, and 5LZO-5SDC samples: (**a**) survey spectra (inset: Zn 2p3/2 and Zn 2p1/2 peaks), (**b**) Ce 3d core-level spectra, and (**c**) O 1s core-level spectra; and (**d**) Raman spectra for LZO, SDC, 5LZO–5SDC, and the 5LZO-5SDC treated in hydrogen and oxygen at 550 °C.

Whereafter, XPS was utilized to probe surface properties of the LZO, SDC, and 5LZO-5SDC samples. Figure 9a shows the survey spectra. It can be clearly seen that there are characteristic peaks of Sm, Zn, Ce, O, C and Li in 5LZO-5SDC. The existence of the characteristic peak of lithium is also confirmed, indicating that Li has been completely incorporated into the crystal lattice of ZnO through occupying an interstitial site or a substitution site of Zn. The peaks of Zn 2p3/2 and Zn 2p1/2 for LZO and 5LZO-5SDC are compared in the inset of Figure 9a, which show almost same locations but different intensities. This is due to the fact that LZO has lower excitation sensitivity of photoelectrons to the X-ray than SDC, leading to less detected photoelectrons than SDC in the 5LZO-5SDC sample. In order to identify the chemical state, the core energy level spectra of Ce 3d and O 1s are deconvoluted by Gaussian functions and Shirley background. Figure 9b shows the coexistence of Ce^4+^ and Ce^3+^ ions on the surface of SDC and 5LZO-5SDC samples. The curve is deconvolved with six peaks (v, v″, v‴, u, u″ and u‴) corresponding to Ce^4+^ and four peaks (v_0_, v′, u_0_ and u′) attributed to Ce^3+^ [34,51]. In our XPS results, the ratio analysis of Ce ions in different oxidation states was assessed by integrating the corresponding peak areas. The result found the 5LZO-5SDC sample has a larger ratio of Ce^3+^, which shows 3% enhancement as compared with that of SDC, as calculated by the Avantage software (version 4.67; Thermo Fisher Scientific, Gloucester, UK, 2013). Generally, the existence of Ce^3+^ is related to the formation of oxygen vacancies. Therefore, the larger Ce^3+^ content in the composite sample means more oxygen vacancies on the surface. Besides, Figure 9c shows the O1s spectra for the three samples. The O 1s core energy level spectra shown in the figure include lattice oxygen (O_α_), oxygen defects (O_δ_), and surface chemisorbed oxygen species (O_β_) adsorbed on surface oxygen vacancies. The binding energy at 531.8–533.7 eV can be allocated to the surface oxygen species (O_β_), the binding energy at 530.8–531.0 eV can be assigned to oxide defects (O_δ_), and that at 528.3–529.6 eV is attributed to lattice oxygen (O_α_). After calculation, it is found that the relative ratio of (O_β_ + O_δ_)/O_α_ increases significantly from the two individual materials to the composite sample, indicating that the surface oxygen vacancies and oxygen defects in the composite are enriched. Since the surfaces of LZO and SDC form hetero-interfaces in the composite, such enrichment of oxygen vacancies and oxide defects should mainly take place at hetero-interface regions, leading to remarkable interface ionic conduction of the LZO-SDC electrolyte.

Moreover, Figure 9d displays the Raman spectra with visible (532 nm) excitation laser wavelengths (λ_ex_) of the LZO, SDC, raw 5LZO-5SDC samples, and the 5LZO-5SDC treated under fuel cell operating condition for 2 h, respectively. According to the peaks of 438 cm^−1^ and 461 cm^−1^, it can be concluded that there is no obvious reaction between LZO and SDC. The refractive index of the main Raman active mode of the symmetric oxygen lattice Ce-O tensile vibration (F2g) in fluorite-type CeO_2_ is at about 460 cm^−1^. In our case, the peak is located at around 461 cm^−1^ (denoted as peak α). Compared with CeO_2_, the synthesized SDC and 5LZO-5SDC samples showed a lower energy shift. In addition, since the substitution of Sm^3+^ ions in cerium oxide will result in the form of oxygen vacancies, a new characteristic pattern (represented as peak β) appears at 554 cm^−1^. The intensity ratio of the peak β reflecting the oxygen vacancy and the peak α representing the F2g mode of the fluorite structure is calculated from their peak area and recorded as A_β_/A_α_, indicating the relative oxygen vacancy concentration and defect type of doped cerium oxide [52]. In the figure, compared with SDC, the A_β_/A_α_ of 5LZO-5SDC has a certain degree of increase, which indicates a higher oxygen vacancy concentration. Worth noting is that, after hydrogen and oxygen treatment at 550 °C, the ratio of A_β_/A_α_ experiences a significant increase, which shows that the LZO-SDC composite can produce higher oxygen vacancy concentration in the actual fuel cell operating condition. Even though SDC itself may also gain more oxygen vacancies in reducing atmosphere, this behavior is almost negligible, as SDC is always apt to be partially reduced by H_2_ to induce high electronic conduction [53]. Thus, combined with the XPS result, the increased oxygen vacancy concentration of the composite should be predominantly a result of hetero-interface formation between LZO and SDC.

### 3.5. Stability

**Figure 10:**
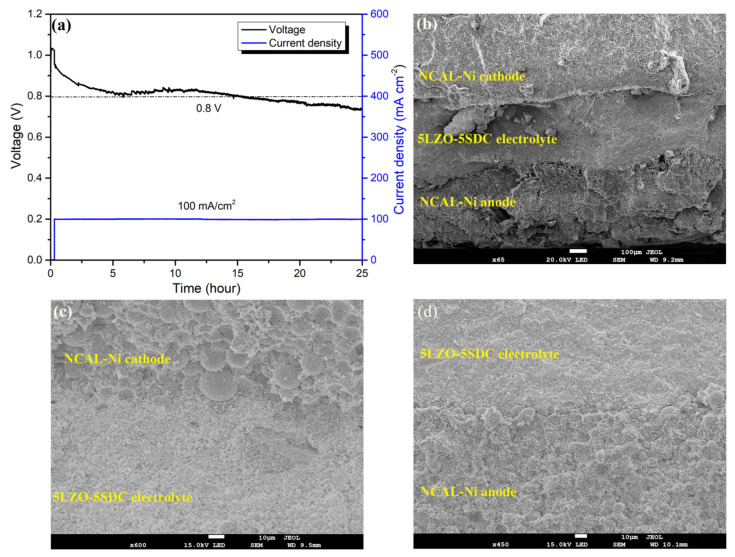
(**a**) Stability result of a 5LZO-5SDC SOFC at 550 °C and (**b**) cross-sectional view of the 5LZO-5SDC SOFC after stability measurement; Enlarged cross-sectional SEM images focusing on the (**c**) anode/electrolyte interface and (**d**) electrolyte/cathode interface to show the morphology of electrodes and electrolyte.

Finally, the long-term durability of the best-performing 5LZO-5SDC SOFC was evaluated at 550 °C under a galvanostatic mode with stationary current density of 100 mA cm^−2^. It was found the composite electrolyte and the SOFC device can basically retain stable during a 25 h stability test. As shown in Figure 10a, the working voltage of the cell showed a degradation during the initial period because of the activation process, and subsequently gradually approached a stable state of around 0.8 V in the following 15 h. This means the developed 5LZO-5SDC composite hold potential to fill the role of electrolyte for practical LT-SOFCs. After 20 h operation, non-negligible voltage degradation can be seen, with a reduction to 0.74 V. This issue is mostly a result of largely increased resistance of the used fuel cell fixture, as we found apparent rust on the surface of the steel chamber of the fixture after 22 h. Moreover, Figure 10b provides an SEM image to illustrate the cross-section view of the 5LZO-5SDC SOFC after stability measurement, in which, three components respectively corresponding to NCAL cathode, 5LZO-5SDC electrolyte, and NCAL anode can be clearly distinguished. The 5LZO-5SDC electrolyte with a thickness of ~320 μm is gas-tight and well adhered to the two porous electrodes, presenting a good mechanical strength in the device. Although the cross section shows rough morphology owing to the squeezing damage when scissoring fuel cell pellet for SEM characterization, it is still observed that the fuel cell device maintains a good three-layer structure without obvious delamination after long-term operation. Figure 10c,d provide two enlarged cross-sectional SEM images focusing on the anode/electrolyte interface and electrolyte/cathode interface of the cell, respectively. The electrolyte layer is not as dense as conventional SOFC electrolyte, but still presents good gas-tightness. The two electrodes show somewhat different morphology, as the anodic NCAL particles are reduced into metallic particles by H_2_, while the cathodic NCAL remain unchanged in air. From Figure 10c,d, it is also noticed that the cathode is less porous than the anode in spite of the fact the same materials were used. This can be majorly attributed to the unfavourable gradual oxidation of Ni-foam on the air side that might take place at 550 °C, which would deteriorate the porous structure of cathode and cause sluggish mass transfer of the cathode reaction. We think this is also an underlying factor leading to the decreased durability of our 5LZO-5SDC SOFC after 20 h operation. Thus, the electrode component and sample fixture should be modified in our follow-up work to demonstrate more potential of 5LZO-5SDC.

## 4. Conclusions

In summary, a new type of composite LZO-SDC with various compositions have been developed in this work for LT-SOFC electrolytes application. XRD and SEM results confirm that LZO and SDC coexist in the composite without chemical interaction and the particles are evenly distributed in the composite material. When applied as the electrolytes in LT-SOFCs, the 5LZO-5SDC was found to be the optimal sample, exhibiting a peak power density of 713 mW cm^−2^ with high OCV of 1.042 V at 550 °C. On the basis of polarization curves and electrical studies, it is found the LZO-SDC composite has much higher conductivity in a reducing atmosphere, and the 5LZO-5SDC sample achieved a remarkable hybrid H^+^/O^2−^ conductivity of 0.16 S cm^−1^ at 550 °C with dominated H^+^ conduction, which is much higher than that of pure LZO. Further investigation in terms of LZO/SDC interface characterization manifests that the massive oxygen vacancies are induced at the hetero-interface between LZO and SDC, which gives rise to the fast ionic transport of 5LZO-5SDC. Finally, the stability of the 5LZO-5SDC SOFC was assessed and a stable operation for ~20 h was observed. Our study thus suggests an effective way to promote the electrolyte functionality of semiconductor electrolyte materials for developing LT-SOFCs.

## Figures and Tables

**Table 1:** The equivalent-circuit analysis results performance of the LZO-SDC SOFCs with various compositions at 550 °C, respectively.

	*R_0_* (Ω cm^2^)	*R_1_* (Ω cm^2^)	*Q_1_* (F cm^−2^)	*n_1_*	*C_1_* (F)	*R_2_* (Ω cm^2^)	*Q_2_* (F cm^−2^)	*n_2_*	*C_2_* (F)	*R_P_* = *R_1_* + *R_2_*
3LZO-7SDC	0.0939	0.0478	1.2320	0.5641	0.1380	0.2083	2.7020	0.7340	2.1940	0.2561
5LZO-5SDC	0.0782	0.0905	1.8600	0.6617	0.7480	0.1574	0.9454	0.4784	0.1185	0.2479
7LZO-3SDC	0.1372	0.1174	0.5378	0.5230	0.0430	0.3634	1.6400	0.6752	1.2790	0.4808

**Table 2:** The comparison of 5LZO-5SDC fuel cell with previously reported fuel cells using the same NCAL-Ni electrodes at 550 °C in terms of *R_o_* and *R_p_*.

Cell Electrolyte	*R_o_* (Ω cm^2^)	*R_p_* (Ω cm^2^)	Reference
LZO	0.1773	0.3809	[14]
SDC	0.1842	1.1032	[34]
STO-SDC	0.1074	0.2915	[34]
LSCF-SCDC	0.32	>2	[22]
NCAL-NSDC	0.1	~0.4	[23]
5LZO-5SDC	0.0782	0.2479	This work

**Table 3:** The DC electrical conductivities of 5LZO-5SDC at 450–550 °C in ambient air, pure nitrogen, and pure hydrogen, and the corresponding activation energies.

	550 °C	500 °C	450 °C	*E_a_* (eV)
$\sigma_{H_{2}}$ (S cm^−1^)	0.028	0.01585	0.0055	0.693
$\sigma_{Air}$ (S cm^−1^)	1.5 × 10^−4^	7.5 × 10^−5^	2.625 × 10^−5^	0.829
$\sigma_{N_{2}}$ (S cm^−1^)	9.66 × 10^−5^	6.188 × 10^−5^	2.25 × 10^−5^	0.557

## Data Availability

The data that support the findings of this study are available from the corresponding author on request.
